# *Spiroplasma ixodetis* Infections in Immunocompetent and Immunosuppressed Patients after Tick Exposure, Sweden

**DOI:** 10.3201/eid2808.212524

**Published:** 2022-08

**Authors:** Johannes Eimer, Louise Fernström, Louise Rohlén, Anna Grankvist, Kristoffer Loo, Erik Nyman, Anna J. Henningsson, Mats Haglund, Viktor Hultqvist, Johanna Sjöwall, Christine Wennerås, Thomas Schön

**Affiliations:** Visby County Hospital, Visby, Sweden (J. Eimer, E. Nyman);; Linköping University, Linköping, Sweden (L. Fernström, L. Rohlén, K. Loo, A.J. Henningsson, M. Haglund, V. Hultqvist, J. Sjöwall, T. Schön);; Kalmar County Hospital, Kalmar, Sweden (L. Fernström, L. Rohlén, K. Loo, M. Haglund, V. Hultqvist, T. Schön);; Sahlgrenska University Hospital, Gothenburg, Sweden (A. Grankvist, C. Wennerås);; Jönköping County Hospital, Jönköping, Sweden (A.J, Henningsson);; Linköping University Hospital, Linköping, Sweden (J. Sjöwall, T. Schön);; University of Gothenburg, Gothenburg (C. Wennerås)

**Keywords:** Spiroplasma ixodetis, bacteria, Anaplasma phagocytophilum, ticks, tick-borne infections, doxycycline, immunocompetent patients, immunosuppressed patients, Sweden

## Abstract

We report 2 cases of *Spiroplasma ixodetis* infection in an immunocompetent patient and an immunocompromised patient who had frequent tick exposure. Fever, thrombocytopenia, and increased liver aminotransferase levels raised the suspicion of anaplasmosis, but 16S rRNA PCR and Sanger sequencing yielded a diagnosis of spiroplasmosis. Both patients recovered after doxycycline treatment.

Acute febrile illness after tick bites may be caused by various agents (e.g., *Borrelia* spp., tick-borne encephalitis virus*, Babesia* spp., *Rickettsia* spp., *Neoehrlichia mikurensis*, *Anaplasma phagocytophilum*). *Spiroplasma ixodetis* was initially described as a cause of neonatal cataract and uveitis (*1*,*2*). Systemic infections caused by other *Spiroplasma* spp. have been reported in 3 immunocompromised patients (*3*–*5*).

*Spiroplasma* spp. are intracellular organisms that belong to the class Mollicutes, which include *Mycoplasma* spp. These bacteria have a single-layer cell membrane, cannot be visualized by Gram staining, require special substrates for growth, and can be diagnosed by genetic methods (*6*). Plants, insects, and ticks are known reservoirs (*7*). *S. ixodetis* was initially reported in *Ixodes pacificus* ticks from Oregon, USA (*8*), and has since been detected in many arthropod species, including *Ixodes ricinus* ticks in several countries in Europe, but not yet in Sweden (*9*,*10*). We report *S. ixodetis* infections in an immunocompetent patient and an immunocompromised patient after tick exposure in Sweden.

## The Study

Oral and written informed consent were obtained from the 2 patients. Case-patient 1 was an 81-year-old previously healthy woman who sought care at the emergency department of Kalmar County Hospital (Kalmar, Sweden) in July 2021 because of a 3-day history of fever (temperature up to 39°C) and mild headache. She reported frequent tick exposure in southeastern Sweden but no history of opportunistic infections or immunosuppressive diseases or treatments that would have compromised immune defenses. She was admitted because of clinical suspicion of anaplasmosis.

**Figure 1:**
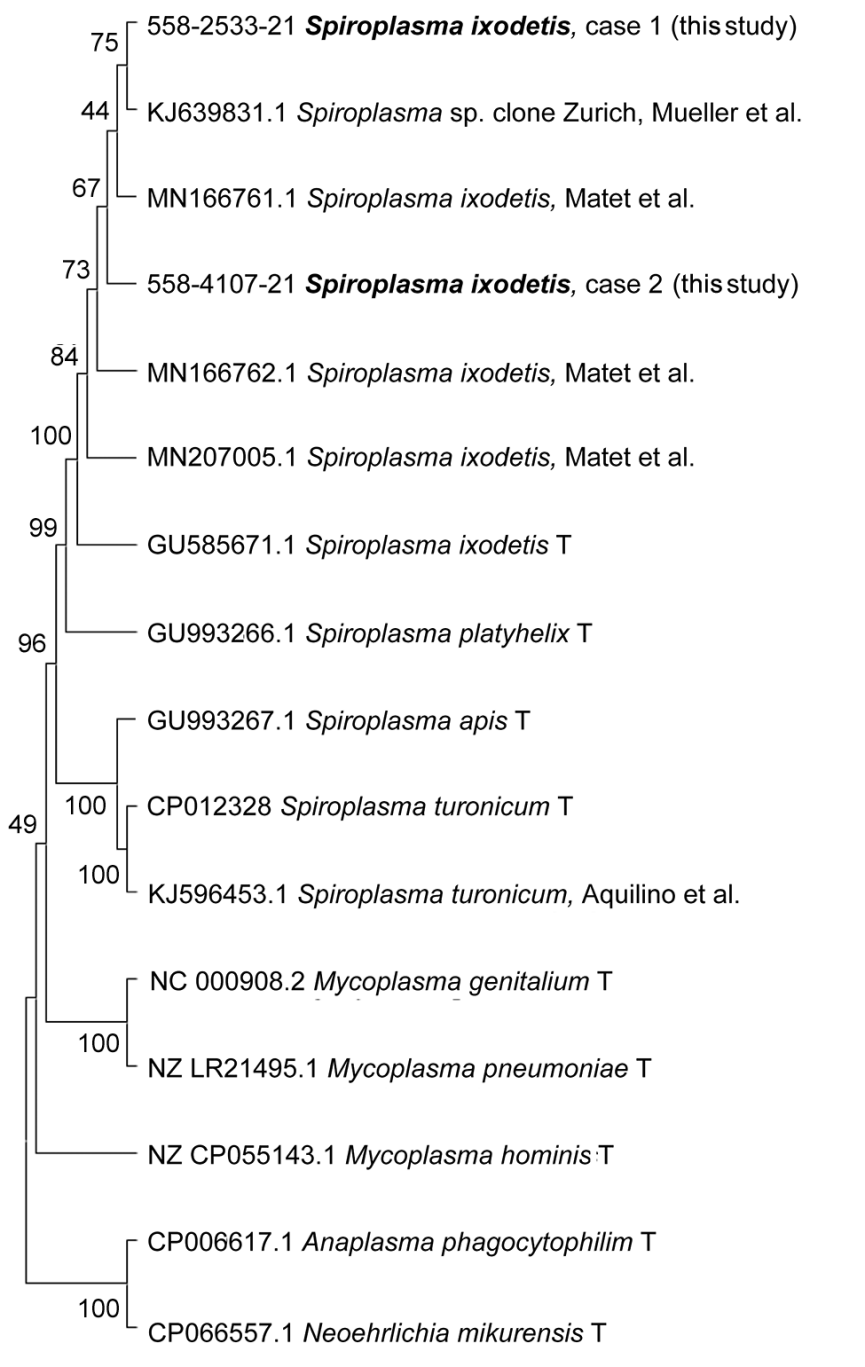
*Spiroplasma ixodetis* infections in immunocompetent and immunosuppressed patients after tick exposure, Sweden. Neighbor-joining tree based on partial 16S rRNA sequences of clinical isolates of *Spiroplasma* spp*.,* other members of the family Mollicutes (*Mycoplasma* spp.), and tickborne bacterial pathogens of the family Anaplasmataceae (*Anaplasma phagocytophilum* and *Neoehrlichia mikurensis*). Type strains are indicated by T, and clinical samples from this study are indicated in bold. Percentage values of replicate trees in which the associated taxa clustered together in the bootstrap test (1,000 replicates) are shown next to the branches. Evolutionary distances were computed by using the Kimura 2-parameter method and are in the units of number of base substitutions per site. Evolutionary analyses were conducted by using MEGA11 (https://www.megasoftware.net).

Blood tests showed thrombocytopenia and increased levels of C-reactive protein (CRP) and alanine aminotransferase (ALT) (Table). Real-time PCR specific for *A. phagocytophilum* (*11*) and *N. mikurensis* (*12*) on EDTA-anticoagulated whole blood showed negative results. However, 3 days after admission, 16S rRNA PCR and Sanger sequencing analysis (Appendix) identified *S. ixodetis* that had 99.72% sequence homology with a reference strain of *S. ixodetis* (GenBank accession no. MN166761) (Figure 1). The *S. ixodetis* sequence has been deposited in GenBank (accession no. OL636349).

**Table:** Results of analysis for *Spiroplasma ixodetis* infections in immunocompetent and immunosuppressed patients after tick exposure, Sweden*

Analysis	Reference value	Case-patient 1, immunocompetent†					Case-patient 2, immunosuppressed‡			
		D0	D1	D2	D16		D0	D2	D4	D25
Clinical chemistry										
Blood										
Hemoglobin, g/L	134–170	143	162	NT	145		114	118	110	108
Leukocytes, × 10^9^ cells/L	3.5–8.8	4.2	3.0	NT	6.4		6.1	6.4	8.8	6.4
Lymphocytes, × 10^9^ cells/L	1.1–3.5	NT	NT	NT	1.9		0.3	NT	0.6	NT
Neutrophils, × 10^9^ cells/L	1.6–5.9	NT	NT	NT	3.7		4.9	NT	7.7	NT
Platelet count, × 10^9^/L	140–350	150	118	NT	287		47	43	41	159
Plasma										
ALT, μkat/L	<1.1	1.6	2.1	NT	0.85		3.82	8.18	13.34	0.48
Creatinine, μmol/L	45–90	66	73	NT	72		75	90	149	71
CRP, mg/L	<5	59	59	38	<1		197	158	164	<3
Vital signs										
O_2_ saturation, %	95‒100	95	95	97			95	97	97	NT
Respiratory rate, breathes/min	12‒16	20	18	20			20	24	20	NT
Blood pressure, mm Hg	90/60‒120/80	108/50	94/69	114/56			117/72	119/66	120/74	NT
Heart rate, beats/min	60‒100	66	90	79			73	74	94	NT
Temperature, °C	37	38.6	37.4	36.2			37.1	39.9	36.4	NT
Immunology/microbiology										
Serum										
IgG1, g/L	4.0–10	NT	NT	NT	7.0		NT	NT	NT	NT
IgG2, g/L	1.7–7.9	NT	NT	NT	3.4		NT	NT	NT	NT
IgG3, g/L	0.1–0.85	NT	NT	NT	0.48		NT	NT	NT	NT
IgG4, g/L	0.03–2	NT	NT	NT	0.15		NT	NT	NT	NT
IgA, g/L	0.9–4.5	NT	NT	NT	3.7		NT	2.4	NT	NT
IgG, g/L	6.7–15	NT	NT	NT	12		NT	10.6	NT	NT
IgM, g/L	0.3–2.1	NT	NT	NT	2.7		NT	0.80	NT	NT
Blood culture	NR	‒	NT	NT	NT		‒	NT	NT	NT
Urine culture	NR	‒	NT	NT	NT		‒	NT	NT	NT
COVID-19 PCR/rapid test	NR	‒	NT	NT	NT		‒	NT	NT	NT

*Day 0 indicates day of admission. ALT, alanine aminotransferase; COVID-19, coronavirus disease; CRP, C-reactive protein; D, day; NT, not tested; NR, not relevant; ‒, negative. †Case-patient 1 had negative PCR results for *Anaplasma* spp. and *Neoerlichia* spp. at admission to the emergency department. ‡Case-patient 2 had negative PCR results in serum for *Anaplasma* spp. and *Neoerlichia* spp. at admission, as well as negative IgG results for *Anaplasma *spp. Results were positive in serum for IgG against *Borrelia burgdorferi* and tick borne encephalitis virus, which were compatible with past infection. Molecular testing (DNA) for Epstein-Barr virus, cytomegalovirus, adenovirus, and parvovirus B19 showed negative results. Test results were negative for antinuclear antibodies and antineutrophil cytoplasmic antibodies, and urine sediment test result was unremarkable.

A slight increase in IgG convalescent-phase titer against *A. phagocytophilum* was observed (from 1:160 to 1:320 during a 4-week interval; reference titer <1:160). However, the result was disregarded because of the negative *A. phagocytophilum* PCR result at admission.

The fever decreased promptly when oral doxycycline treatment (100 mg 2×/d) was initiated. The patient was discharged, and treatment was continued for a total of 10 days. Upon follow-up, the patient had recovered and had no remaining laboratory result abnormalities (Table). Total serum immunoglobulins, including IgG subclasses, were within reference ranges, and a follow-up blood sample was negative by 16S rRNA PCR.

Case-patient 2 was a 76-year-old man who had insulin-dependent type 2 diabetes and Crohn’s disease who had been given infliximab maintenance therapy. He was on a prednisolone taper after an exacerbation of his inflammatory bowel disease. The patient sought care at the emergency department of Visby County Hospital (Visby, Sweden) in October 2021 for a 2-week history of spiking fevers and fatigue. He reported multiple tick bites throughout summer and had been given penicillin V for erythema migrans. No other focal signs or symptoms were reported.

**Figure 2:**
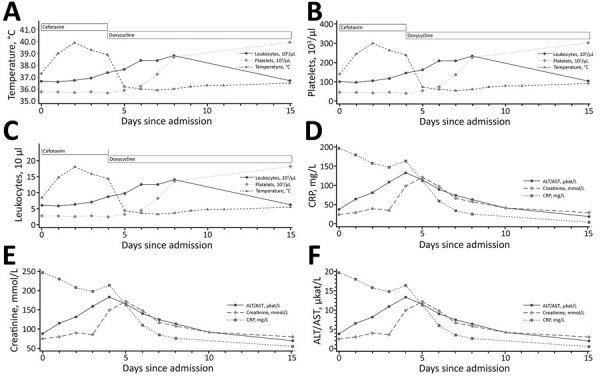
Clinical course of *Spiroplasma ixodetis* infection in an immunocompromised 76-year-old man (case-patient 2) after tick exposure, Sweden. ALT, alanine aminotransferase; CRP, C-reactive protein.

At admission, blood tests showed pancytopenia with predominant thrombocytopenia and increased CRP and ALT levels (Table). Empirical treatment with intravenous cefotaxime was started. Aminotransferase levels quadrupled during the next 4 days, and acute kidney injury developed (Figure 2). Results of routine examinations, such as blood cultures, serologic tests, and molecular tests, were inconclusive (Table).

Given the progressive clinical picture, cefotaxime was replaced on day 5 by doxycycline (100 mg 2×/d), which resulted in return of liver and kidney functions to reference values within 1 week and improved clinical condition (Figure 2). The patient was discharged after 11 days; doxycyline treatment was continued for 21 days. A serum sample (1 mL) from day 4 was analyzed for *N. mikurensis* and *A. phagocytophilum* by PCR and unbiased bacterial 16S rRNA sequencing. Analysis identified *S. ixodetis* in serum that had 99.86% sequence homology with a reference strain of *S. ixodetis*. The patient sequence has been deposited in GenBank (accession no. OL636350) (Figure 1). The patient remained well 6 weeks after symptom onset and had no residual abnormal laboratory results.

## Conclusions

We report 2 cases of systemic *S. ixodetis* infection that were presumably acquired by tick bites in southeastern Sweden. This organism has not been reported in *Ix. ricinus* ticks from Sweden, but *A. phagocytophilum*, *N. mikurensis, Rickettsia* spp., and *Babesia* spp. are endemic tickborne microorganisms that may cause febrile illness. However, thrombocytopenia and increased levels of liver enzymes rarely occur in neoehrlichiosis (*13*). *A. phagocytophilum* infections can cause thrombocytopenia and increased levels of liver enzymes, but are an uncommon cause of fever in Scandinavia, and *Babesia* spp. affects primarily severely immunocompromised persons (*14*).

A case of human *Spiroplasma* infection was reported in Germany during 2002 and involved a 4-month-old premature child who had unilateral cataract and uveitis (*1*). Three case reports have described systemic infections caused by *Spiroplasma* spp. The first case involved a 73-year-old woman from Spain who had selective IgM deficiency, rheumatoid arthritis, fever, myalgia, headache, and bilateral conjunctivitis; she was receiving tumor necrosis-α and interleukin-6 inhibitors (*4*). *S. turonicum* was identified by 16S rRNA PCR performed on blood cultures. Her fever was unresponsive to cefuroxime but resolved after she received doxycycline and levofloxacin for 2 months.

The second case involved a 70-year-old woman from Switzerland who had diffuse abdominal pain and fatigue. She was a lung transplant recipient and was afebrile. Laboratory analysis showed, consistent with our cases, thrombocytopenia and increased liver enzyme levels. Liver biopsy and blood samples analyzed by 16S rRNA PCR identified *Spiroplasma* sp. that had 98.2% homology with *S. ixodetis*, referred to as *Spiroplasma* sp. Zurich (*5*). The patient received doxycycline and azithromycin for 2 months and slowly recovered.

The third case involved a 40-year-old man who had X-linked agammaglobulinemia and febrile polyarthritis. Blood and synovial fluid cultures grew small bacterial colonies unidentifiable by routine methods, but 16S rRNA PCR identified *S. apis* (*3*). He recovered after a 12-week course of levofloxacin and doxycycline.

In reports on systemic *S. ixodetis* infection, doxycycline was prescribed in combination with either levofloxacin or azithromycin (*2*). In our study, the patients showed improvement after doxycycline monotherapy and were cured without relapse, supporting the notion that doxycycline is effective against *S. ixodetis* infections. The previously described systemic infections were caused by other species of *Spiroplasma* (*S. turonicum*, *S. apis*, and *Spiroplasma* sp. Zurich). The route of transmission was unclear except for the *S. apis* case, for which a hornet sting was the plausible route of transmission. In contrast, the *S. ixodetis* patients we describe were most likely infected via tick bites acquired in the coastal areas of southeastern Sweden, including the islands of Öland and Gotland.

Our study suggests an association between tick exposure and human *S. ixodetis* infection. Previous case reports of human *Spiroplasma* infection have been associated with an immunocompromised state, either in the form of immature eyes of newborns or conditions requiring immunosuppressive treatment. We report a systemic *Spiroplasma* infection in an apparently immunocompetent person. However, immunosenescence of the aged immune system might have compromised innate or acquired immune defenses (*15*).

In conclusion, we report 2 case-patients who had *S. ixodetis* infection and acute febrile illness after tick exposure. Treatment with doxycycline was successful. This finding shows the clinical utility of unbiased 16S rRNA analysis for correct diagnosis and treatment, as well as its potential for identifying novel pathogens in the febrile host. We are developing a *Spiroplasma*-specific PCR that might increase sensitivity of detection. *S. ixodetis* is an emerging pathogen that should be considered in patients with febrile illness after tick exposure.

Appendix Additional information on *Spiroplasma ixodetis* infections in immunocompetent and immunosuppressed patients after tick exposure, Sweden.

## References

[1] Lorenz B, Schroeder J, Reischl U. First evidence of an endogenous *Spiroplasma* sp. infection in humans manifesting as unilateral cataract associated with anterior uveitis in a premature baby. Graefes Arch Clin Exp Ophthalmol. 2002;240:348–53. 10.1007/s00417-002-0453-3 12073057

[2] Matet A, Le Flèche-Matéos A, Doz F, Dureau P, Cassoux N. Ocular *Spiroplasma ixodetis* in newborns, France. Emerg Infect Dis. 2020;26:340–4. 10.3201/eid2602.191097 31793858 PMC6986854

[3] Etienne N, Bret L, Le Brun C, Lecuyer H, Moraly J, Lanternier F, et al. Disseminated *Spiroplasma apis* infection in patient with agammaglobulinemia, France. Emerg Infect Dis. 2018;24:2382–6. 10.3201/eid2412.180567 30457541 PMC6256403

[4] Aquilino A, Masiá M, López P, Galiana AJ, Tovar J, Andrés M, et al. First human systemic infection caused by *Spiroplasma.* J Clin Microbiol. 2015;53:719–21. 10.1128/JCM.02841-14 25428150 PMC4298541

[5] Mueller NJ, Tini GM, Weber A, Gaspert A, Husmann L, Bloemberg G, et al. Hepatitis from *Spiroplasma* sp. in an immunocompromised patient. Am J Transplant. 2015;15:2511–6. 10.1111/ajt.13254 25832127

[6] Harne S, Gayathri P, Béven L. Exploring *Spiroplasma* biology: opportunities and challenges. Front Microbiol. 2020;11:589279. 10.3389/fmicb.2020.589279 33193251 PMC7609405

[7] Cisak E, Wójcik-Fatla A, Zając V, Sawczyn A, Sroka J, Dutkiewicz J. Spiroplasma - an emerging arthropod-borne pathogen? Ann Agric Environ Med. 2015;22:589–93. 10.5604/12321966.1185758 26706960

[8] Tully JG, Rose DL, Yunker CE, Carle P, Bové JM, Williamson DL, et al. *Spiroplasma ixodetis* sp. nov., a new species from *Ixodes pacificus* ticks collected in Oregon. Int J Syst Bacteriol. 1995;45:23–8. 10.1099/00207713-45-1-23 7857803

[9] Binetruy F, Bailly X, Chevillon C, Martin OY, Bernasconi MV, Duron O. Phylogenetics of the *Spiroplasma ixodetis* endosymbiont reveals past transfers between ticks and other arthropods. Ticks Tick Borne Dis. 2019;10:575–84. 10.1016/j.ttbdis.2019.02.001 30744948

[10] Olsthoorn F, Sprong H, Fonville M, Rocchi M, Medlock J, Gilbert L, et al. Occurrence of tick-borne pathogens in questing *Ixodes ricinus* ticks from Wester Ross, Northwest Scotland. Parasit Vectors. 2021;14:430. 10.1186/s13071-021-04946-5 34446082 PMC8393815

[11] Wass L, Grankvist A, Mattsson M, Gustafsson H, Krogfelt K, Olsen B, et al. Serological reactivity to *Anaplasma phagocytophilum* in neoehrlichiosis patients. Eur J Clin Microbiol Infect Dis. 2018;37:1673–8. 10.1007/s10096-018-3298-3 29948363 PMC6133046

[12] Grankvist A, Sandelin LL, Andersson J, Fryland L, Wilhelmsson P, Lindgren P-E, et al. Infections with *Candidatus* Neoehrlichia mikurensis and cytokine responses in 2 persons bitten by ticks, Sweden. Emerg Infect Dis. 2015;21:1462–5. 10.3201/eid2108.150060 26197035 PMC4517700

[13] Sjöwall J, Kling K, Ochoa-Figueroa M, Zachrisson H, Wennerås C. *Neoehrlichia mikurensis* causing thrombosis and relapsing fever in a lymphoma patient receiving rituximab. Microorganisms. 2021;9:2138. 10.3390/microorganisms9102138 34683459 PMC8537581

[14] Madison-Antenucci S, Kramer LD, Gebhardt LL, Kauffman E. Emerging tick-borne diseases. Clin Microbiol Rev. 2020;33:e00083–18. 10.1128/CMR.00083-18 31896541 PMC6941843

[15] Rodrigues LP, Teixeira VR, Alencar-Silva T, Simonassi-Paiva B, Pereira RW, Pogue R, et al. Hallmarks of aging and immunosenescence: Connecting the dots. Cytokine Growth Factor Rev. 2021;59:9–21. 10.1016/j.cytogfr.2021.01.006 33551332

