# Supplementary material for: Spiroplasma ixodetis Infections in Immunocompetent and Immunosuppressed Patients after Tick Exposure, Sweden
Source: Emerg Infect Dis. 2022 Aug;28(8):1681–5. doi: 10.3201/eid2808.212524 (PMC9328919; doi:10.3201/eid2808.212524)
Supplement: Appendix — Additional information on Spiroplasma ixodetis infections in immunocompetent and immunosuppressed patients after tick exposure, Sweden. [file 21-2524-Techapp-s1.pdf]

Article DOI: https://doi.org/10.3201/eid2808.212524

Spiroplasma ixodetis Infections in Immunocompetent and Immunosuppressed Patients after Tick Exposure, Sweden

Appendix

DNA Extraction

Bacterial DNA was extracted and concentrated from 1.0–1.5 mL samples of plasma or serum by centrifugation at 16,000 × *g* for 5 min. DNA was extracted from 400 μL of the resulting pellet by using the MagLEAD Extraction Robot (Precision System Science, https://www.pss.com.jp) and the MagDEA Dx SV Isolation Kit I (Precision System Science) according to the manufacturer's instructions and using a 50-μL elution volume.

16S rRNA PCR and Sanger Sequencing

A segment of the bacterial 16S rRNA gene was amplified by using the forward primer SSU1mod 5′-CGG CGT GCC TAA CAC ATG CAA GTC G-3′ and the reverse primer 806R, 5′-GGA CTA CCA GGG TAT CTA AT-3′, which are complementary to conserved regions in the 5′ half of the 16S rRNA gene (V1–V4). Master mix and amplification procedure were as described (*1*). For sequencing, PCR products of the expected size (766 bp) were isolated by using gel electrophoresis, removed from the gel, purified by using the Qiaquick Gel Extraction Kit (QIAGEN, https://www.qiagen.com/), and cycle sequenced by using BigDye Terminator v3.1 (Applied Biosystems, https://www.thermofisher.com). Sequences were analyzed by using the ABI PRISM 3130/3500 Genetic Analyzer (Applied Biosystems). After editing of the DNA sequence, it was compared with sequences in GenBank by using BLAST, NCBI (*2*).

References

1. Welinder-Olsson C, Dotevall L, Hogevik H, Jungnelius R, Trollfors B, Wahl M, et al. Comparison of broad-range bacterial PCR and culture of cerebrospinal fluid for diagnosis of community-acquired bacterial meningitis. Clin Microbiol Infect. 2007;13:879–86. PubMed https://doi.org/10.1111/j.1469-0691.2007.01756.x

2. Skovbjerg S, Welinder-Olsson C, Kondori N, Kjellin E, Nowrouzian F, Wold AE, et al. Optimization of the detection of microbes in blood from immunocompromised patients with haematological malignancies. Clin Microbiol Infect. 2009;15:680–3. PubMed https://doi.org/10.1111/j.1469-0691.2009.02796.x
